# The Essential Oils; Their Value and Use in Dentistry

**Published:** 1900-09-15

**Authors:** H. Prinz

**Affiliations:** St. Louis, Mo.


THE
### DENTAL REGISTER.

Vol. LIV. September 15, 1900. No. 9.

### THE ESSENTIAL OILS; THEIR VALUE AND USE IN DENTISTRY.

BY H. PRINZ, D.D S., ST. LOUIS, MO.

Essential, etherial, volatile or distilled oils, as they are termed, are mostly derived by distillation from plants. Chiefly, the oils are found in the fruit, flowering part, or the bark of the plants. Occasionally the plant may produce two or even three different oils in its various parts (orange tree). They have a strong odor and taste, and are used to a large extent in perfumery and medicine as flavoring agents, while dentistry relies chiefly upon them for their antiseptic and obtundent qualities.

Essential oils do not belong to a definite chemical group. Most of them are composed of a mixture of two or more principles, one being a hydrocarbon, known as a terpene, and the other being an oxygenated body, such as an aldehyde, (oil of cassia) an acid, (oil of cloves) a phenol, (oil of thyme) an esther, (oil of wintergreen) etc. It is this oxygenated body upon which the value of the oil as an antiseptic or obtundent depends. The oils are usually slightly colored, yellowish, brown, red, green, etc., but this color, it is believed, is due to some small amount of vegetable matter present. Sometimes the stills or original containers of the oil imparts the color. The green color of the oil of cajuput may be traced to the copper stills or copper canisters.

Essential oils are easily vaporized and undergo some decomposition with age, by absorbing oxygen. They become darker in color and thick, depositing resinous precipitates. The oils are soluble in alcohol, ether, chloroform, fatty oils, etc. Agitated with water they form a milky mixture from which the oil will soon separate, imparting its odor and taste to the water.

In dentistry the essential oils are mostly employed as antiseptics, obtundents, solvents, etc. The most important use is as antiseptics. Their value as germicidal agents depends largely upon their volatility, which, according to Cushny, "enables them to penetrate readily into protoplasm and to lessen its vitality by acting as foreign bodies. In addition, they are nearly related to the benzol series, the members of which are all antiseptics and protoplasm poisons." No doubt, their toxic properties are due to the oxygenated body present. This fact is demonstrated by the action of the oil upon bacteria in culture medium. The more concentrated the oil, viz., the more active constituent present, the more rapid its action as an antiseptic.

*Oil of Cassia.*—"Oleum Cinnamomi, a volatile oil distilled from Cassia Cinnamom."—U. S. Ph. This oil comprises a number of varieties. Oil of cassia and oil of cinnamon are synonymous; the oil of cassia proper is imported from China, while the oil of cinnamon comes from Ceylon. There is a cinnamon leaf oil found in commerce which, according to Schimmel & Co.'s reports, is almost pure eugenol, resembling therefore very much the oil of cloves. The value of oil of cassia as an antiseptic depends solely upon the amount of cinnamic aldehyde present. A good oil should contain at least from 80 to 85 percent thereof. It has been stated by a writer upon this subject in a recent paper that "this oil as found in commerce is not so potent an antiseptic by about one-half as was the cassia obtained ten years ago." This statement is erroneous. From the best authentic sources upon essential oils the writer has obtained this statement: "Oil of cassia as found in the market to-day is far superior to an article which was sold some ten years ago. Oil of cassia is exclusively handled according to the percentage of cinnamic aldehyde." A series of experiments have substantiated this conclusion. The artificial oil of cassia is pure cinnamic aldehyde. The normal oil of cassia has one great objection; its persistent use in root canal treatment will discolor the tooth. This is due to the deposition of colored resenous substances, the terpenes, in the dental tubuli. The so-called "two-fold, free from terpenes" or the synthetical oil should therefore only be used for such work.

*Oil of Cloves.*—"Oleum Caryophylli, a volatile oil distilled from Cloves."—U. S. Ph. The value of oil of cloves depends upon the eugenol present. Eugenol, a phenol-like body, resembling an acid, has superior qualities as an obtundent, is antiseptic and almost non-irritating. It penetrates deeply into dental tissues, more so if they are previously dehydrated by means of absolute alcohol and the hot air blast. Eugenol is especially useful in the treatment of the living or inflamed pulp, as this organ takes very kindly to such non-irritating applications. Quite a number of other essential oils owe their property chiefly to the engenol present, such as cinnamon leaf oil, oil of bay, oil of allspice, etc.

*Oil of Eucalyptus.*—"Oleum Eucalypti, a volatile oil distilled from fresh leaves of Eucalyptus Globules and some other species of Eucalyptus."—U. S. Ph. Eucalyptol, a neutral body, is the active constituent of the distilled oil; it is merely a purified oil of eucalyptus. Eucalpytol has been much lauded in medicine as having antimalarial properties. In fact, countries where malaria is persistently prevailing have been greatly benefited by the cultivation of the eucalyptus tree, as for instance the Campagna, a swampy district near the city of Rome, Italy. The antiseptic properties of the oil in regard to root treatment are very limited. It has practically no obtunding influence upon dentin. Oil of eucalyptus and other members of the same group, such as oil of cajuput, oil of myrtle (myrtol) possess the property of dissolving gutta-percha, the eucalyptol being the acting factor. Cajuputol of the oil of cajuput and cineol of the oil of myrtle are chemically identical with eucalyptol. It is claimed that a solution of gutta-percha in any of these three oils is superior for root fillings or setting of crowns.

*Oil of Thyme.*—"Oleum Thymi, a volatile oil distilled from the leaves and flowering tops of Thymus Vulgaris."—U. S. Ph. Druggists list two varieties of the oil, the white and the red; the white being a purified product of the crude, red oil. Oftentimes a crude oil is imported from France under the name of oil of thyme which is oil of origanum (wild marjoram.) The value of oil of thyme depends upon thymol, a phenol, occurring in the volatile oils of thymus vulgaris, monarda punctata (horsemint), and carum adjowan (Indian caraway). Thymol is a colorless, crystaline substance, having the agreeable odor of the oil and a pungent, aromatic taste. It melts at about 112° F., is freely soluble in alcohol, ether, chloroform, fatty oils, but slightly in water (1.1200). When triturated with camphor, menthol or chloral it liquifies. The melted thymol dissolves in liquified carbolic acid. Thymol in its antiseptic action is almost equal to carbolic acid, and in some respects even better. It is not so caustic as the former, as it is more destructive of putrefactive substances and more persistant in its action, it at once recommends itself as a useful agent for treating putrescent root canals. A mixture of equal parts of liquified carbolic acid, and thymol is superior to carbolic acid alone and far superior to any of the essential oils. Thymol is very penetrating, combined with other agents it has been used by Paquet with great success for the preservation of anatomical specimens, and its superior germicidal power has been recently sufficiently demonstrated by the British government in India in the treatment of the bubonic plague. Its great obtunding and astringent qualities make it a reliable dressing for imflamed pulps, as recommended by Miller years ago. It is curious to note that this agent, in spite of its superiority in many ways over the other essential oils, is comparatively little used by American dentists, while its established value is recognized by the profession in Europe.

*Oil of Wintergreen.*—"Oleum Gaultheræ, a volatile oil distilled from the leaves of Gaultheria Procumbens, consisting almost entirely of Methyl Salicylate and nearly identical with volatile oil of Betula."—U. S. Ph. Both the oil of wintergreen and the oil of sweet birch have proven to be valueless in the treatment of pulpless teeth. An artificial oil, pure methyl salicylate, shares the same fate. These oils are stimulating and astringent and are much in favor as flavors for oral specialities.

*Menthol.*—"A Stearoptene (having the character of a secondary alcohol), obtained from the official oil of peppermint or from Japanese or Chinese oil of peppermint."—U. S. Ph. Oil of peppermint is little used as a dental antiseptic. Its most persistent odor forbids its frequent application. Menthol possesses anodyne and anesthetic properties, which are useful in connection with other remedies in the treatment of the living pulps.

The testing of essential oils by adding a few drops of the diluted oils upon some cryptogamic plants transplanted from the mouth into a test tube, and to draw positive conclusions from such experiments is misleading. If we expect to derive exact results from experimental work, we should apply the oils to infected material taken from a gangrenous pulp and subject the whole to conditions similar to those found in the root canals. And still we must always bear in mind that a test tube is *not* the human body and that laboratory experiments have to be verified by clinical results before we may report positive facts.

In treating teeth with gangrenous pulps, the most important work lies in the mechanical cleansing of the root canals. "One should guard against the error that mistakes in manipulation can be equalized by the use of a strong antiseptic." (Miller.)

The root canals of human teeth are lined with a thin layer of bony material which to some extent resembles cementum. This layer is almost impenetrable to microorganisms, and in rare cases one may find by examining microscopical sections here and there a few isolated bacilla. "I am convinced that by thorough cleansing of the root canal up to the apical foramen we need not pay much attention to the dentinal tubuli." (Miller.)

Chemical agents have been recommended for such work which will remove the contents of a canal and sterilize it at the same time, viz.: Callahan's sulphuric acid methods; Schreier's alloy of potassium and sodium; potassium and sodium hydroxide, sodium peroxide (Kirk), etc.

It is not our object to criticise these methods; we will confine our remarks to the essential oils used in this connection. After the canal is cleansed, serumal exudations from the apical foramen have to be disinfected and drained off. The question now arises, What antiseptic shall we use, an essential oil or carbolic acid? The secretions of an infected root canal—and, for that matter, any infected wound—consist largely of albuminous fluids, mingled with pus, microbes, ptomaines, etc. The object of an antiseptic dressing is to render the micro-organisms and their products inert, thereby disengaging the antiseptic present from the dressing and taking up the exudations. If we employ an agent which is soluble in water or in serum (viz., carbolic acid), we will accomplish the desired effect more quickly than by using an insoluble drug such as the essential oils. Essential oils upon a cotton dressing confined in a root canal will be very slowly disengaged, as oil and water have no affinity for each other. The watery exudations prevent the oils from readily disseminating. If we employ a mixture of carbolic acid and thymol, the action of carbolic acid is materially assisted by the less soluble thymol, which, as stated above, is a powerful antiseptic, causing little or no irritation.

According to Cushny, it is believed at present that a mixture of drugs belonging to the benzol series is more strongly antiseptic relatively than the individual substances. A solution of equal parts of thymol and carbolic acid is therefore more efficient than the corresponding proportion of either alone.

This principle may explain the much lauded Black's 1-2-3 mixture. By taking test tubes filled with a solution of egg albumen, into each of which is immersed cotton fibers saturated respectively with carbolic acid, carbolic acid and thymol, and an essential oil, one will find the above described phenomena roughly illustrated. Carbolic acid soon dissolves, thymol and carbolic acid requires more time, while the oil will only be disengaged by vigorous shaking. The oily cotton fibers will not take up any of the albumen solution. The objection has been raised that carbolic acid will coagulate albumen; essential oils will not. This is true, but the coagulum is of such a nature as not to prevent the further penetration of the acid, which has been amply demonstrated (York, Kirk, Trueman). The action of carbolic acid in precipitating albumens may be rather compared to alcohol, in which the proteid is precipitated, not because an insoluble compound is formed, but because of a change in the nature of the solvent (Curberry). Metallic antiseptics, such as corrosive sublimate, are rendered insoluble by albumen. The addition of an acid or sodium chloride will overcome this objection. A solution of one and one-half grains of mercuric chloride with one grain of citric or tartaric acid in four ounces of peroxide of hydrogen (which is slightly acid in itself) can be highly recommended for washing out putrid root canals. It often becomes necessary to increase the antiseptic strength of the liquid preparations by adding more powerful drugs. The iodin compounds, such as iodoform, aristol, iodol, sozoiodol, nosophene, etc., have gained great favor with the dentists. The germicidal action of these chemicals depends largely upon the liberation of the iodin atom present, and, as the essential oils will chemically combine with free iodin, iodin preparations should therefore be mixed with pure carbolic acid.

It is probably worth while mentioning that the crystalline carbolic acid should be liquefied with pure glycerin rather than with alcohol or water, for dental purposes.

The very interesting question arises now, How do these iodin compounds act? This question has been for a long time a source of much controversy in medical literature, more so in regard to the action of iodoform. Iodoform is the strongest antiseptic of the iodin group with the possible exception of the tri-chloride of iodin, but unfortunately this latter is strongly acid in reaction. Iodoform is not a germicide in comparison with carbolic acid or mercuric chloride. Pathogenic germs may be grown in culture media, mixed with large quantities of iodoform; it is not even sterile. In spite of these laboratory experiments it has steadily grown in favor with the surgeons, and is still recognized as the best agent for checking suppuration. According to modern views, suppuration is not so much due to the bacteria present, but rather to their waste products, i.e., the ptomaines. If iodoform is brought in contact with water and oxygen, a continuous liberation of iodin slowly takes place (Binz), a condition met with in root canals, and it is this continuous liberation of the iodin atom which renders bacteria and their products inert. Thus it checks secretion and combines with newly granulating tissue, acting somewhat as an astringent. The action of iodoform is therefore a modified iodin action. Peabody and Kirchner tried to obtain similar results by applying iodoform in vapor form, but they found it to be too irritating to the tissues. It is the gradual liberation of iodin which makes iodoform a power in surgery and only its penetrating and persistant odor has checked its career in dentistry.

Aristol, a copyrighted name for thymol iodide and an almost odorless substitute for iodoform has been much lauded by dentists for root treatment (Kirk). While iodoform contains about 95 percent. of available iodin, aristol has only about 45 percent. It seems to have little influence upon lower forms of life (Neisser) and does not check suppuration very readily.

The other named iodin compound have gained little favor with dentists or are not long enough upon the market to be properly judged as to their usefulness.

In epitomizing the foregoing thoughts we draw the following conclusions:

1. The value of essential oils as antiseptic agents for the treatment of infected root canals is greatly over-estimated.

2. Carbolic acid in the early stages and carbolic acid and thymol, equal parts, for treatment after suppuration has ceased, is superior to the essential oils.

3. Carbolic acid in combination with some iodin compounds, preferably iodoform, is a reliable agent for the treatment of suppuration in infected root canals.

4. Certain essential oils have value as dental obtundents and as therapeutical agents for the treatment of the early stages of inflammation of the pulp or as vehicles for pulp capping material.

BIBLIOGRAPHY.

Cushny, Pharmacology; Hare, Therapeutics; Burchard, Dental Pathology; Miller, The Micro-organisms of the Human Mouth; The U. S. Dispensatory; Schimmel & Co.'s Semi-Annual Reports; Merck's Index; Binz, Arzneimittellehre; Miller, Conservative Zahnheilkunde; Dental Digest; Correspondenzblatt fuer Zahnærzte; L'Odontologie.

